# Predictors of the early pneumatic displacement effect of submacular haemorrhage secondary to polypoidal choroidal vasculopathy

**DOI:** 10.1038/s41433-026-04535-9

**Published:** 2026-05-25

**Authors:** Fang Zheng, Jingjie Xu, Yabo Han, Yufeng Xu, Shijing Wu, Jingliang He, Ailing Sui, Ye Liu, Li Zhang, Panpan Ye, Zhitao Su, Yongwei Guo, Lei Liu, Li Huo, Xiaoyun Fang

**Affiliations:** 1 Eye Center, Zhejiang University School of Medicine Second Affiliated Hospital, Hangzhou, China; 2 Department of Ophthalmology, Yuyao People’s Hospital of Zhejiang Province, Ningbo, China; 3 Guangdong Eye Institute, Department of Ophthalmology, Guangdong Provincial People’s Hospital, Guangzhou, China; 4 Department of Electronic Engineering, Tsinghua University, Beijing, China

**Keywords:** Predictive markers, Macular degeneration

## Abstract

**Purpose:**

To identify baseline predictors of early submacular haemorrhage (SMH) displacement following intravitreal injection of C3F8, recombinant tissue plasminogen activator (rt-PA), and ranibizumab in patients with polypoidal choroidal vasculopathy (PCV).

**Methods:**

In this retrospective case-control study, swept-source OCT scans were analysed before and one week after treatment. SMH displacement was classified as incomplete (Group 1) or complete (Group 2) based on fundus photography and OCT. Clinical characteristics and OCT-derived parameters were evaluated for associations with displacement status.

**Results:**

Fifty-one eyes from 51 PCV patients were included. All eyes demonstrated some degree of SMH displacement after treatment, with 33 eyes showing incomplete displacement (residual SMH under the fovea: 80–768 μm) and 18 eyes showing complete displacement. Group 2 had better baseline visual acuity, and baseline LogMAR BCVA was negatively correlated with displacement grade. Group 2 also exhibited larger initial SMH volume and lower contrast-to-noise ratio (CNR), both associated with greater displacement. Although baseline PED volumes were similar, PED regression was more pronounced in Group 2.

**Conclusion:**

Complete SMH displacement was associated with better baseline vision, lower CNR, larger haemorrhage volume, and greater PED regression. Baseline BCVA, CNR, and SMH volume may serve as predictive factors for early SMH displacement following treatment.

## Introduction

Submacular haemorrhage (SMH) is a vision-threatening complication of polypoidal choroidal vasculopathy (PCV). The incidence of SMH is greater in PCV than in typical neovascular age-related macular degeneration (AMD) [1]. It can be the first sign in treatment-naïve patients, or it can even happen to patients who have already undergone anti- vascular endothelial growth factor (VEGF) treatment. If left untreated, the photoreceptor will suffer from irreversible damage [2, 3], and the vision outcomes are poor [4, 5]. Several treatment options are effective for removing SMH from macula [6–8], and clinical studies have been performed to compare their effectiveness and safety [9–12]. One randomised controlled trial showed that pars plana vitrectomy (PPV) together with subretinal injection of recombinant tissue plasminogen activator (rt-PA), gas tamponade, and intravitreal Ranibizumab injection did not yield superior benefits for SMH compared with pneumatic displacement (PD) combined with intravitreal rt-PA, and Ranibizumab injection [13]. The cost-effectiveness, easy-to-perform, and minimally invasive features of PD are advantages that cannot be ignored when choosing treatment.

Clinicians are investigating factors influencing the displacement effect of SMH and vision recovery. Smaller areas with good vision at presentation benefit the most from PD for a group of patients with SMH secondary to AMD [14]. Central pigment epithelial detachment (PED) thickness has been shown to be the most important factor associated with one-year visual outcomes of treatment with one session of PD for SMH secondary to PCV [15]. Previous studies have also demonstrated that a useful predictor for early displacement of SMH is contrast-to-noise ratio (CNR), an objective evaluation of signal intensity of structures on optical coherence tomography (OCT) images, after both intravitreal SF6 gas injection without the use of rt-PA or anti-VEGF agents [16] and PPV together with subretinal rt-PA injection [17]. However, the OCT parameters used in these studies are based on only a single B-scan through the fovea. Other information might be missed in only one image.

With the advantages of swept-source OCT (SS-OCT), widefield three-dimensional cube scans provide more information regarding the whole volume of SMH and PEDs [18]. What is more, unlike the aforementioned study [16], the currently widely used PD technique is combined with simultaneous use of intravitreal injection of rt-PA and anti-VEGF agents. This approach might influence the results, since rt-PA may facilitate subretinal clot dissolution and anti-VEGF therapy targets the underlying PCV.

Therefore, we investigated the predictors of early displacement of SMH after intravitreal injection of C3F8, rt-PA, and Ranibizumab in this study.

## Methods

Patients diagnosed with SMH secondary to PCV who underwent PD treatment followed by the same procedure from August 2022 to July 2025 were enrolled in this retrospective case-control study at Eye Center, Second Affiliated Hospital of Zhejiang University. This study was approved by the institutional review board of the hospital (NO.20220637). Written informed consent was obtained from all participants. Basic information, including age, sex, current anticoagulation medications, smoking history, alcohol consumption, previous ocular surgeries, and duration of symptoms, was collected. All patients underwent comprehensive eye examinations before and after surgery, including best corrected visual acuity (BCVA), intraocular pressure, fundus photos (CLARUS 500, Carl Zeiss, Dublin, USA or Daytona P200T, Optos PLC, Dunfermline, UK), and SS-OCT 3D cube scans (BM-400K BMizar, TowardPi Medical Technology, Beijing, China). The 12 mm × 12 mm or 24 mm × 20 mm SS-OCT scans were selected based on the size of SMH in order to cover the whole SMH. BCVA at the most recent visit and the occurrence of vitreous haemorrhage after PD were also recorded. Eyes with no SS-OCT 3D cube scans taken before and one week after the surgery were excluded. Eyes with extensive haemorrhage exceeding the 24 mm × 20 mm scan area were excluded.

### Intravitreal injection

First, adequate aqueous humour drainage of 0.3–0.4 ml after povidone-iodine sterilisation was performed. An amount of 0.025 ml (25 μg) rt-PA (Actilyse®, Boehringer Ingelheim Pharma GmbH & Co.KG) was injected intravitreally using a thirty-gauge needle. Then, 0.05 ml (0.5 mg) of Ranibizumab (Novaritis Pharma Schweiz AG) was injected intravitreally. Finally, 0.2–0.3 ml of pure C3F8 gas was injected. Additional aqueous humour drainage was performed as needed to adjust the intraocular pressure after gas injection. Intravenous infusion of 250 ml of mannitol was administered immediately following the surgery, except in cases where intraocular pressure (IOP) normalised after adequate aqueous humour drainage, most commonly observed in pseudophakic eyes with a deep anterior chamber. Topical anti-glaucoma medications were initiated, if IOP remained elevated two hours post-injection. The patients were asked to remain prone for three days after the treatment.

### Displacement grading

**Fig. 1:**
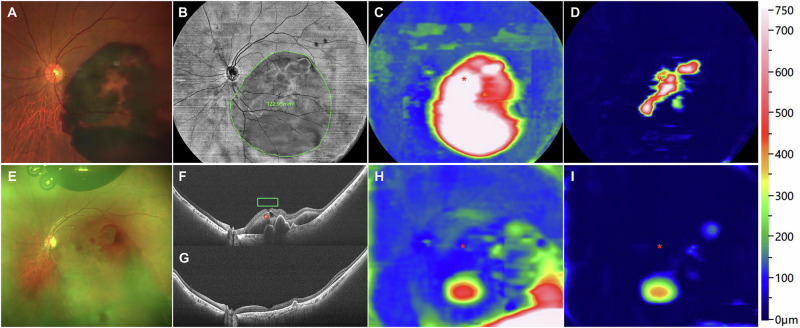
A male in his sixties with treatment-naïve PCV in the left eye demonstrating the complete displacement of SMH after pneumatic displacement treatment. **A** The baseline colour fundus picture showing subretinal haemorrhage together with PED in the macula. **B** The 24 mm × 20 mm enface SS-OCT image with the boundary from OPL to BM showing the outlining of the SMH area (green line). **C** The retinal thickness map generated from the same cube scan of (**B**) with the boundary from OPL to BM. The red asterisk indicating the location of the fovea. **D** The PED volume map generated from the same cube scan of (**B**) with the boundary from RPE to BM. **E** The colour fundus photo taken one week after the surgery showing the displacement of SMH out of the macula. **F** The OCT B-scan through the fovea taken before the surgery showing subretinal haemorrhage and PED. The area inside the red circle was chosen to be the foreground and the area inside the green box was chosen to be the background to calculate CNR. **G** The OCT B-scan through the fovea taken one week after the surgery showing no remaining haemorrhage under the fovea, a small PED, and a small amount of residual SMH temporal to the macula. **H** The retinal thickness map generated at one week after the surgery with the boundary from OPL to BM demonstrating the displacement of the SMH. **I** The retinal thickness map generated one week after the surgery with the boundary from RPE to BM demonstrating the regression of PED.

The displacement grading was based on both colour photos and OCT images taken one week after surgery. For eyes with apparent displacement of SMH on colour fundus photos, the OCT B-scans passing through the fovea were checked to determine whether any residual SMH remained beneath the fovea. If SMH was found on the fovea OCT B-scan, the case was defined as incomplete displacement (Group 1), even if colour fundus photos revealed that SMH was well displaced. Complete displacement (Group 2) was defined as SMH displaced out of the macula with no residual SMH under the fovea (Fig. 1).

### Measurements

The SMH area was measured on the enface OCT image with the segmentation from outer plexiform layer (OPL) to Bruch’s membrane (BM) using the built-in tool by outlining the haemorrhage boundaries (Fig. 1B). The height of SMH under the fovea was measured using the built-in calliper tool. The measurement was defined as the vertical distance from the apex of the haemorrhage to BM, with the measurement line drawn perpendicular to BM. The volume of SMH, were calculated based on the retinal map with the boundaries from OPL to BM after adjusting the segmentation manually (Fig. 1C). The volume of PED was calculated based on the retinal map with the boundaries from retinal pigment epithelium (RPE) to BM (Fig. 1D, I). The horizontal OCT B-scan image taken through the fovea was used to calculate the CNR. A small area of haemorrhage beneath the fovea was chosen as the foreground, and a certain area of the vitreous cavity above the fovea was chosen as the background (Fig. 1F). We calculated the CNR using open-source software (ImageJ, National Institutes of Health, Bethesda, MD) in the same way as a previous Japanese study [17]. All segmentation lines, including OPL, RPE, and BM, were carefully reviewed and manually corrected when necessary before quantitative analysis. The measurements including SMH area, the height of SMH under fovea and CNR were performed by two experienced ophthalmologists (HYB and HJL) who were blinded to the displacement effect, and average values were taken for the final statistical analysis.

### Statistical analysis

All data are presented as mean ± standard deviation or median [Q1, Q3], where Q1 and Q3 represent the first and third quartiles, respectively. Normality of data distribution was assessed using the Shapiro–Wilk test. BCVA values were converted to the logarithm of the minimum angle of resolution (LogMAR) for statistical analysis. Following a previous report [19], eyes with BCVA recorded as counting fingers were assigned an arbitrary LogMAR value of 2.6. Interobserver reliability for measurements obtained by the two ophthalmologists was evaluated using the intraclass correlation coefficient (ICC) based on a two-way random-effects model with absolute agreement. Categorical variables were analysed using Fisher’s exact test, and continuous variables were compared using either the independent-samples *t*-test or Mann–Whitney U test, as appropriate. Paired *t*-tests or Wilcoxon signed-rank tests were used to assess changes in LogMAR BCVA, SMH height beneath the fovea, and PED volume at different time points. Associations between continuous variables were evaluated using Pearson’s correlation coefficient.

Baseline variables that showed significant differences between groups were selected as potential predictors and further analysed for their association with SMH displacement grade using logistic regression modelling. Model calibration was assessed with the Hosmer–Lemeshow test. Discriminatory performance of both the logistic regression model and individual predictors was evaluated using receiver operating characteristic (ROC) curve analysis, with the area under the curve (AUC) calculated to quantify predictive accuracy. A two-sided *P* value < 0.05 was considered statistically significant. All analyses were performed using IBM SPSS Statistics for Windows, Version 26.0 (IBM Corp., Armonk, NY, USA).

## Results

A total of 64 patients were initially included in this retrospective study. Transient IOP elevation within two hours after PD was observed in 47 of 64 patients (23–35 mmHg), which was controlled with short-term topical anti-glaucoma medications and returned to normal by postoperative day one. One patient developed vitreous haemorrhage at the postoperative day three visit and subsequently underwent pars plana vitrectomy, which prevented the acquisition of widefield SS-OCT cube scans and led to exclusion from the analysis. No cases of rhegmatogenous retinal detachment occurred during the study follow-up. Ten patients were excluded due to missing widefield SS-OCT cube scans either at baseline or one week after surgery. Two patients were excluded for extensive haemorrhage exceeding the 24 mm × 20 mm scan area, which precluded accurate OCT measurements. Therefore, 51 eyes from 51 patients were enrolled in the final analysis. The ICCs for SMH area, the height of SMH under fovea and CNR measurements between two ophthalmologists were 0.86 (95% CI, 0.76–0.91; *P* = 0.005), 0.88 (95% CI, 0.66–0.95; *P* = 0.004) and 0.79 (95% CI, 0.63–0.88; *P* = 0.001).

**Fig. 2:**
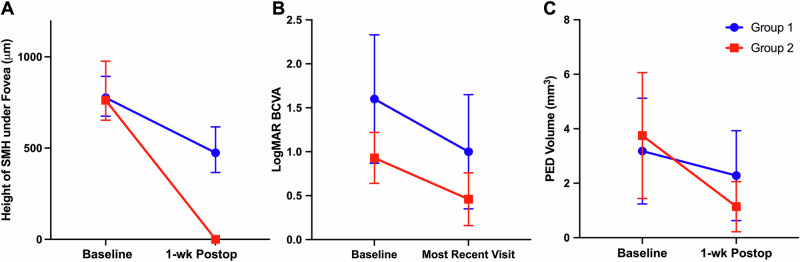
Changes in subfoveal SMH height, LogMAR BCVA, and PED volume before and after treatment. **A** Height of SMH under the fovea. **B** LogMAR BCVA. **C** PED volume.

Following treatment, all eyes exhibited some degree of SMH displacement: the SMH of 33 eyes was incompletely displaced (Group 1), and the SMH of 18 eyes was completely displaced (Group 2). The mean height of the residual SMH under the fovea was 276 μm [149.5–476.5], ranging from 80 μm to 768 μm in Group 1 (Fig. 2A). There was no difference between the two groups in the age, sex, use of anticoagulant medication, alcohol consumption, smoking history, or history of cataract surgery (Table 1). Five patients in Group 1 and one patient in Group 2 had previously received anti-VEGF treatments. Notably, one of the five patients in Group 1 developed SMH on the third day following the third ant-VEGF injection. All the other patients were treatment-naïve for PCV. Three patient in Group 1 developed recurrent haemorrhages, including two cases of subretinal haemorrhage occurring one month after surgery and one case of breakthrough VH two weeks postoperatively, caused by RPE tear confirmed during vitrectomy. One patient developed a recurrent subretinal haemorrhage in Group 2 (Table 1).

**Table 1:** Demographic and ophthalmological characteristics of patients with incomplete (Group 1) and complete (Group 2) SMH displacement.

	Group 1 (*N* = 33)	Group 2 (*N* = 18)	
Age (years)	65.24 ± 9.69	67.89 ± 9.49	*P* = 0.352
Male (*n*)	18 (54.5%)	9 (50.0%)	*P* = 0.778
Duration of symptoms (days)	10 [5.0–14]	10 [7.25–16]	*P* = 0.757
Use of anticoagulant medicine (*n*)	5 (15.2%)	1 (5.6%)	*P* = 0.405
Alcohol history (*n*)	9 (27.3%)	2 (11.1%)	*P* = 0.288
Smoking history (*n*)	13 (39.4%)	7 (38.9%)	*P* = 1.000
IOL (*n*)	4 (12.1%)	5 (27.8%)	*P* = 0.249
Previous anti-VEGF treatment (*n*)	5 (15.2%)	1 (5.6%)	*P* = 0.405
Rebleeding (*n*)	3 (9.1%)	1(5.6%)	*P* = 1.000
LogMAR BCVA			
Baseline	1.60 ± 0.73	0.93 ± 0.29	*P* = 0.005*
Most recent BCVA	1.00 ± 0.65	0.46 ± 0.30	*P* = 0.005*
Improvement	0.59 ± 0.85	0.47 ± 0.31	*P* = 0.439
CNR	4.90 ± 1.35	3.60 ± 1.21	*P* = 0.009*
Height of SMH under fovea (μm)			
Baseline	776 [675–893]	762.5 [652.75–976.25]	*P* = 0.708
1 week after treatment	276 [149.5–476.5]	0	*P* = 0.000*
Reduction	474 [366.5–616]	762.5 [652.75–976.25]	*P* = 0.007*
SMH area (mm^2^)	49.85 ± 28.34	78.91 ± 35.11	*P* = 0.012*
Volume of SMH at baseline (mm^3^)	33.14 ± 23.95	53.62 ± 27.99	*P* = 0.008*
Volume of PED (mm^3^)			
Baseline	3.18 ± 1.94	3.75 ± 2.31	*P* = 0.352
1 week after treatment	2.28 ± 1.65	1.14 ± 0.92	*P* = 0.009*
Reduction	0.90 ± 0.85	2.61 ± 2.15	*P* = 0.003*
Reduction in proportion	0.32 ± 0.25	0.63 ± 0.25	*P* = 0.001*

*IOL* intraocular lens, *Anti-VEGF* anti-vascular endothelial growth factor, *BCVA* best-corrected visual acuity, *CNR* contrast-to-noise ratio, *SMH* submacular haemorrhage, *PED* pigment epithelial detachment. *P< 0.05.

The duration of symptoms did not differ significantly between the two groups (Table 1). However, patients in Group 2 presented with markedly better vision at their first visit. Both groups experienced improvement in BCVA after treatment (Fig. 2B). The interval between the most recent visit and surgery was 57 days [30–109] in Group 1 and 61 days [35–120] in Group 2 (*P* = 0.930). Patients in Group 2 had a significantly lower CNR of SMH at baseline (4.90 ± 1.35 vs. 3.60 ± 1.21; *P* = 0.009), and there was no correlation between baseline BCVA and CNR (r = 0.22; *P* = 0.127). The area and volume of SMH were 49.85 ± 28.34 mm² vs. 78.91 ± 35.11 mm² (*P* = 0.012) and 33.14 ± 23.95 mm³ vs. 53.62 ± 27.99 mm³ (*P* = 0.008), respectively, indicating a larger haemorrhage burden in Group 2. However, no significant difference was observed in the height of SMH beneath the fovea: median 776 μm [675–893] in Group 1 vs. 762.5 μm [652.75–976.25] in Group 2 (*P* = 0.708). Baseline PED volume was comparable between groups (3.18 ± 1.94 mm³ vs. 3.75 ± 2.31 mm³; *P* = 0.352). Following treatment, both groups showed a reduction in PED volume, with Group 2 demonstrating a more pronounced decrease (32 ± 25% vs. 63 ± 25%; *P* = 0.001) (Fig. 2C).

**Fig. 3:**
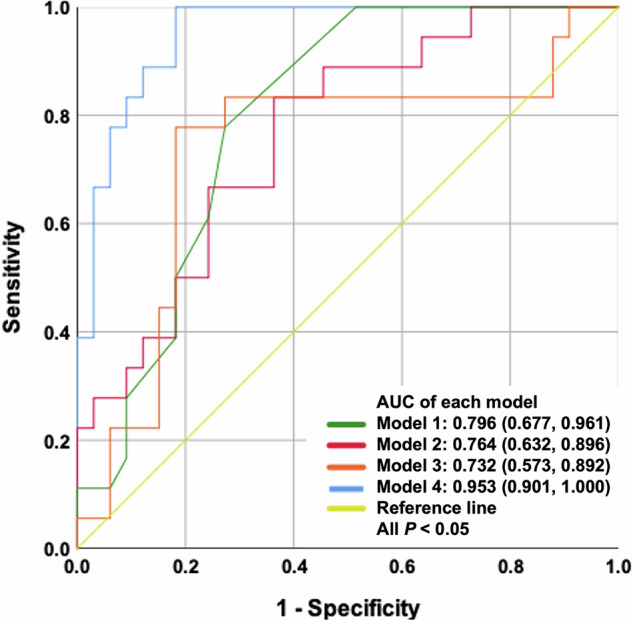
ROC curves for predictive models. The ROC curve for the combined model incorporating baseline LogMAR BCVA, CNR, and SMH volume (Model 4; blue line) is shown alongside curves for baseline LogMAR BCVA alone (Model 1), CNR alone (Model 2), and SMH volume alone (Model 3). The yellow diagonal line indicates the line of identity (AUC = 0.5).

Univariable logistic regression analysis identified baseline LogMAR BCVA, CNR, SMH area, and SMH volume as significant factors associated with SMH displacement (Table 2). Although both SMH area and volume were significant in the univariable analysis, these two parameters demonstrated a higher degree of collinearity (r = 0.803; *P* < 0.001). To maintain model stability and interpretability, only SMH volume was included in the multivariable analysis. The final multivariable logistic regression model identified baseline LogMAR BCVA (OR = 0.014; 95% CI, 0.001–0.381; *P* = 0.011), CNR (OR = 0.421; 95% CI, 0.189–0.937; *P* = 0.034) and SMH volume (OR = 1.076; 95% CI, 1.023–1.131; *P* = 0.004) as independent predictors of complete SMH displacement at one week after treatment. The Hosmer–Lemeshow test indicated no significant deviation between the observed and predicted probabilities (χ² = 2.557; df = 8; *P* = 0.911), suggesting good model calibration. ROC curve analysis demonstrated excellent discriminatory ability of the combined model, with an AUC of 0.953 (95% CI, 0.901–1.000; *P* < 0.001) (Fig. 3).

**Table 2:** Logistic regression analysis of baseline predictors for the grading of displacement at one week.

	Grading of displacement at one week after treatment			
	Univariable analysis		Multivariable analysis	
	OR (95% CI)	*P*	OR (95% CI)	*P*
LogMAR BCVA at baseline	0.082 (0.014–0.489)	0.006*	0.014 (0.001–0.381)	0.011*
CNR	0.446 (0.256–0.777)	0.004*	0.421 (0.189–0.937)	0.034*
SMH area (mm^2^)	1.029 (1.008–1.050)	0.006*	/	/
Volume of SMH at baseline (mm^3^)	1.030 (1.006–1.055)	0.014*	1.076(1.023–1.131)	0.004*

*BCVA* best-corrected visual acuity, *CNR* contrast-to-noise ratio, *SMH* submacular haemorrhage. *P < 0.05.

## Discussion

In this study, we confirmed the effectiveness of PD for treating SMH in patients with PCV and, for the first time, achieved three-dimensional visualisation of both SMH and PED using widefield SS-OCT, enabling a more comprehensive assessment than previously reported. Eyes with complete SMH displacement exhibited better baseline visual acuity, larger haemorrhage volume, and lower CNR compared to those with incomplete displacement, and also showed significantly greater PED regression concurrent with haemorrhage displacement. Beyond confirming baseline BCVA and CNR as established predictors, we identified SMH volume as a novel and valuable predictor of early displacement grade following PD.

In a previous study, 2 out of 16 patients experienced almost no displacement of SMH at one week after simple intravitreal SF6 gas injection [16]. In contrast, in our study, the SMH of all patients was removed to a certain degree, which might be the influence of the rt-PA qualifying the clot. In that prior study, the displacement grade in this study was defined into 3 levels: Grade 0, almost no displacement; Grade 1, mild displacement beyond the arcade; and Grade 2, good displacement outside the fovea. In our cohort, we observed that even when SMH appeared to be displaced outside the macula on colour fundus images, a small residual haemorrhage can be remained beneath the fovea. These cases were difficult to classify using previously published standards. Given that residual subfoveal haemorrhage can continue to damage the overlying retina, we categorised these cases as incomplete displacement, roughly corresponding to Grade 1 in earlier studies, even when the residual SMH height was as low as 80 μm.

The duration of symptoms is a subjective measure that can easily be influenced by patients’ lack of awareness, particularly when the fellow eye has good vision. Baseline visual acuity may therefore serve as an indirect indicator of haemorrhage duration. In our correlation analysis, BCVA was not significantly associated with the self-reported duration of symptoms, which may reflect inaccuracies in patients’ recollection. Experimental studies in rabbits have shown that marked photoreceptor destruction can occur within 24 h of subretinal haemorrhage, with near-complete loss of photoreceptor cells by day 7 [2]. In the current study, there was no significant difference in SMH height under fovea between groups, suggesting that photoreceptors in both groups were subjected to a similar diffusion barrier. The longer photoreceptors are exposed to toxic byproducts of haemorrhage, the greater the degree of vision loss. Unlike the symptom duration reported by patients, BCVA offers a more reliable, objective parameter. In our analysis, baseline LogMAR BCVA emerged as a strong predictor of displacement outcomes, potentially reflecting the combined effect of haemorrhage duration, as blood remains more liquefied in the earlier stages after onset.

CNR is useful for the objective evaluation of signal intensity in regions of interest on OCT images. Because solid materials have high reflectivity in OCT images, a high CNR value would indicate a more solid SMH. A lower CNR of SMH may indicates two potential conditions. First, it may reflect a shorter duration since disease onset, during which the haemorrhage remains largely uncongealed and less solidified. Second, it may suggest that the SMH is admixed with fluid exudated from neovascularisation. Both scenarios contribute to increased ease of blood displacement. Averaging multiple OCT B-scans can affect CNR value [20]. The OCT B-scans we chose for CNR analysis ranged from 12 mm × 12 mm for small-sized SMH to 24 mm × 20 mm for large-sized SMH. Both scan patterns have the same average number, which is two. Therefore, we ruled out influence from different scan patterns. We confirmed that CNR is a useful parameter for predicting the displacement effect.

According to previous reports, the height of SMH is negatively correlated with displacement grade or vision outcomes [14, 16, 21]. The height of SMH was measured in these studies by a single OCT B-scan through the fovea. In our study, we also measured the subfoveal SMH height; however, this parameter alone failed to distinguish between groups, as no significant baseline difference was observed. Instead, we quantified SMH volume, providing a more comprehensive three-dimensional assessment of the haemorrhage, enabled by the advantages of SS-OCT. In cases with a large volume of SMH, gravitational effects cause the accumulated blood shift inferiorly, resulting in no significant difference in subfoveal SMH height between the two groups. Importantly, SMH volume was significantly greater in the complete displacement group and demonstrated a positive correlation with displacement grade in logistic regression analysis. The exact mechanism remains unclear. A possible explanation is that larger haemorrhage volumes may increase the effective contact area between gas bubble and subretinal blood, potentially enhancing the mechanical effect of the gas and facilitating displacement.

PEDs often accompany SMH when it comes to PCV. Central PED thickness was the most important factor associated with the one-year visual outcome of treatment with one session of intravitreal rt-PA, Ranibizumab, and gas injections for SMH secondary to PCV [15]. PEDs have been thought to have a blocking effect on displacing the SMH. However, according to our study, patients in Group 2 had even a larger volume of PED at baseline. This might indicate that the presence of PEDs has little to do with the displacement effect. The flattening of PEDs after pneumatic therapy is not a new finding. Previous studies have reported unexpected PED flattening after PD using rt-PA or subretinal injection of rt-PA [22, 23]. In the current study, we observed the same phenomenon. Furthermore, we found that PEDs in Group 2 regressed to a greater extent. PEDs can be divided into fibrovascular, serous, haemorrhagic, or mixed. Preexisting fibrovascular PEDs are thought to be refractory to PD. For haemorrhagic PEDs, intravitreal rt-PA injection might dissolve not only the subretinal clot but also the conjugated blood under RPE. For either serous PEDs or liquified haemorrhagic PEDs, the pressure from the gas bubble enhances the absorption of the fluid under RPE through choroidal circulation or drainage of the fluid in PEDs via a micro-tear into the subretinal space. The more liquified the content under RPE is, the easier it is to drain or be absorbed. Even though it is difficult to tell the content inside PEDs with massive SMH blocking the OCT signal, the different responses of PEDs in the two groups indirectly confirmed this explanation. PEDs in Group 2 may contain a higher proportion of serous rather than fibrovascular material. In the meantime, the effect of anti-VEGF on PEDs cannot be ignored. We also identified two cases of breakthrough VH caused by RPE tears, which were visualised during PPV and are possibly complications related to PD. More research is needed to investigate the influence of PD on PEDs.

The primary limitation of this study is the absence of post-treatment BCVA data at standardised follow-up intervals, largely due to loss of long-term follow-up in some patients. Consequently, we analysed the most recent BCVA available in the medical records and did not evaluate the effect of PD on visual outcomes. Furthermore, BCVA is influenced by numerous confounding factors, such as prior treatments and lens status, making it difficult to isolate the impact of SMH displacement. Therefore, we could not determine the extent to which early complete SMH displacement affects visual recovery compared to incomplete displacement. Future studies with standardised follow-up protocols and controlled variables are needed to clarify the relationship between early SMH displacement and long-term visual outcomes.

In conclusion, patients who had complete pneumatic displacement of SMH had better initial vision, thicker haemorrhage, and lower CNR at baseline. They also experienced larger PED regression at one week after treatment. These results identify baseline BCVA, CNR, and SMH volume as key, complementary predictors of early displacement outcomes: BCVA reflects symptom duration, CNR indicates the coagulation status of the haemorrhage, and SMH volume represents overall haemorrhage burden and the effective contact area for pneumatic displacement. Together, these findings provide valuable reference data to guide clinical decision-making in the management of SMH.

## Summary

### What was known before

- Pneumatic displacement (PD) combined with intravitreal recombinant tissue plasminogen activator (rt-PA) and Ranibizumab injection has been demonstrated to be effective in the removal of submacular haemorrhage (SMH) in many studies, including a randomised controlled trial. However, the exact predictors of the early displacement effect remain unknown. Although several studies have identified that small SMH height, low contrast-to-noise ratio (CNR), and small pigment epithelial detachment (PED) thickness are contributing factors, previous studies only used a single OCT B-scan through the fovea for measurements, potentially missing other information. Moreover, these previous studies did not always involve rt-PA or anti-VEGF in the treatment method. Therefore, we investigated the predictive factors of early displacement in a group of SMH patients secondary to PCV who underwent uniform treatment.

### What this study adds

- Patients with complete displacement of SMH had better initial vision, larger amounts of haemorrhage, and lower CNR at baseline. They also experienced larger PED volume regression at one week after treatment. The baseline BCVA, CNR and volume of SMH were valuable predictors of early SMH displacement after PD combined with intravitreal recombinant tissue plasminogen activator (rt-PA) and Ranibizumab injection. This study emphasised the importance of 3D visualisation of SMH using widefield swept-source OCT for the first time. The results can assist clinicians making decisions when treating SMH patients.

## Data Availability

The datasets generated during and/or analysed during the current study are available from the corresponding author on reasonable request.
